# Immunotoxicity of polystyrene nanoplastics in different hemocyte subpopulations of *Mytilus galloprovincialis*

**DOI:** 10.1038/s41598-020-65596-8

**Published:** 2020-05-25

**Authors:** Marta Sendra, María Isabel Carrasco-Braganza, Pilar María Yeste, Marta Vila, Julián Blasco

**Affiliations:** 1 CSIC, Spanish National Reference Laboratory for Mollusc Diseases, Institute of Marine Research (IIM), National Research Council (CSIC), 36208 Vigo, Spain; 2 Department of Ecology and Coastal Management, Institute of Marine Sciences of Andalusia (CSIC), Campus Río San Pedro, 11510 Puerto Real, Cádiz Spain; 3 Department of Material Science, Metallurgical Engineering and Inorganic Chemistry, University of Cádiz, Cádiz, Spain; 4 Laboratory of Biochemistry and Molecular Biology, University of Huelva, Huelva, Spain

**Keywords:** Cell death and immune response, Environmental impact

## Abstract

Plastic represents 60-80% of litter in the ocean. Degradation of plastic to small fragments leads to the formation of microplastics (MPs <5 mm) and nanoplastics (NPs <1 µm). One of the most widely used and representative plastics found in the ocean is polystyrene (PS). Among marine organisms, the immune system of bivalves is recognized as suitable to assess nanomaterial toxicity. Hemocyte subpopulations [R1 (large granular cells), R2 (small semi-granular cells) and R3 (small agranular or hyaline cells)] of *Mytilus galloprovincialis* are specialized in particular tasks and functions. The authors propose to examine the effects of different sizes (50 nm, 100 nm and 1 μm) PS NPs on the different immune cells of mussels when they were exposed to (1 and 10 mg·L−1) of PS NPs. The most noteworthy results found in this work are: (i) 1 µm PS NPs provoked higher immunological responses with respect to 50 and 100 nm PS NPs, possibly related to the higher stability in size and shape in hemolymph serum, (ii) the R1 subpopulation was the most affected with respect to R2 and R3 concerning immunological responses and (iii) an increase in the release of toxic radicals, apoptotic signals, tracking of lysosomes and a decrease in phagocytic activity was found in R1.

## Introduction

Currently, the presence of plastics in the marine environment is one of the most serious issues for marine ecosystems^1^. Marine plastic pollution has increased in the last decade due to excessive consumerism, where only 6–26% of the total plastic used is recycled^2^. The main final destiny of plastics is the oceans, which retain between 70–80% of the plastic produced by manufacturing^2^. The areas with higher concentrations of plastics are the subtropical gyres and coastal zones^3,4^. The concentration of plastics in surface water can reach up to 0.5 and 8.5 mg·L^−1^ in the south Pacific gyre and the southern North Sea respectively^5,6^ while the highest concentration recorded in sediment was 32.9 mg·kg^−1^ in Artic sediment^7^.

The plastics found on the surface have a lower density than marine water such as: expanded polystyrene (EPS), low-density polyethylene (LDPE), high-density polyethylene (HDPE), polypropylene (PP), Polyethylene terephthalate (PET), Polyamide (PA), polystyrene (PS) polymethil methacrylate (PMMA); (going from lower to higher densities). Among the plastics used on the market, PS is one of the most widely used for diverse functions such as: for food, industrial packing, cutlery, building insulation, medical products and toys^8^. Moreover, PS is the fourth most abundant plastic in the ocean as microplastics (MPs) and therefore also nanoplastics (NPs)^9^.

MPs and NPs are smaller than 5 mm and 1 µm respectively^8,10^, and they are found in the natural environment originating from primary or secondary sources^11^. Therefore, the MPs and NPs directly released into the environment from manufacturing products of have a primary origin. However, most of the plastics coming from the land and boats are found as macroplastics and mesoplastics (>25 and <25 mm respectively);^2^. These plastics degrade when exposed to environmental agents such as UV, waves and wind into smaller pieces of plastic until reaching the nanometer scale^12^. Knowing the characteristics of these plastics such as the: size, zeta potential and coating and behaviour of MPs/NPs (agglomeration/aggregation, eco/biocorona formation and bioavailability) in natural and biological matrices appears more than ever to be essential in ecotoxicological testing^13^.

Plastic particles can provoke negative effects by ingestion and accumulation in marine organisms. Several works are to be found in the literature about the accumulation and effects of MPs in aquatic organisms including holoplankton, meroplankton, microzooplankton, copepods, rotifers, amphipods, lugworms, sea urchins, brine shrimps, fish, bivalves and crabs^14–19^. However, data about the effects of NPs are scarce.

The main marine group used to assess the negative effects of nanomaterials are the bivalves due to their sessile status and capacity to filter large volume of seawater^20^. A recent review proposed mussels as a bioindicator of microplastic pollution because of: their wide distribution, their occupying vital ecological niches, vulnerability to microplastic uptake and the relationship with predators and human health^21^. In the study by Karlsson *et al*. (2017) 770.8 times more plastic item·kg^−1^ was shown to exist in the dry weight of mussel than in the surrounding sediment^22^.

Several researchers have carried out assays in bivalves exposed to MPs. Some of the negative effects cited in the literature are: feeding modification, reproductive disruption, offspring, pseudofaeces production, adherence in mantle and gills, histopathological damage, alteration in antioxidative and detoxification enzymes, downregulation in shell formation genes, embriotoxicity and trophic transfer^13,19,23–29^.

Currently, *in vitro* assays with mussel hemocytes are known to be a powerful tool to assess the effects of nanomaterials and an alternative to conventional methods for a quick but complex screening of the effects and action mechanisms of emergent pollutants^20^. Furthermore, ecotoxicological tests that are easily reproducible under controlled conditions make up the cornerstone of nanotoxicological studies. In tests with nanomaterials, colloidal forces govern the interactions between NPs-cells. These interactions are determined by (i) the intrinsic properties of the nanomaterial such as size, shape, specific surface area, coating and (ii) the suspending medium such as water molecules, salts and polymers^30,31^.

As invertebrates, bivalves rely exclusively on an innate immune system incorporating cellular and humoral components. The immunological system of mussels is efficient against non-self-substances and dead cells, so the immune cells (hemocytes) will react to the input of MPs and NPs^32,33^. Some of the mechanisms involved in immunoregulation are: phagocytosis, activation of the phenoloxydase system, encapsulation, the respiratory burst, nitric oxide production, and the production and release of various microbicidal molecules^34^. Most *in vitro tests* consider hemocytes as a single set of cells. However, several different subpopulations of hemocyte cells are found in mussels. Actually, hemocytes are classified, according to their morphology and histochemistry, as hyalinocytes and granulocytes, and the latter can be further subdivided into eosinophilic granular hemocytes and basophilic granular hemocytes^35^. Mussel hemocyte classification is still a matter of debate; some studies classify hemocytes in two, three and four subpopulations by flow cytometry. The work of Le Foll *et al*. (2010) established three populations. The first one (R1), eosinophilic granulocytes, that are about 10 µm in size with acidic granules of phagosomes and professional phagocytes (also is called R1^36^), The second population (R2), semigranular basic, cells 7 µm [(membrane deformation filopodia condensed cell body and the third population (R3)], hyalinocytes 10 µm, no granules of phagosomes with low phagocytic capacity. According to García-García *et al*. (2008) these same three regions known as R1, R2 and R4 were found; however, an R3 was also recognized, characterized by large semigranular cells. In the present work, the authors are able to recognize the three common subpopulations recorded in Le Foll *et al*., 2010 and García-García *et al*., 2008 without difficulty (see dot plots of hemocytes measured by flow cytometry in Fig. S1). The R3 recorded by García-García *et al*., 2008 was not always easy to discern, so the authors based this study on three subpopulation of hemocytes.

From the hypothesis that subpopulations of mussel hemocytes are involved in different immune defence mechanisms, then different effects on hemocyte subpopulations exposed to NPs might be observed. Furthermore, the different nominal size of NPs might also affect the different subpopulations of mussel hemocytes in distinct and different ways. To test both hypothesis the following goals were proposed: i) to study the effects of different sized PS NPs (50, 100 and 1 µm) on the immune responses of the well-defined three hemocyte subpopulations of *Mytilus galloprovincialis* over time, 3 and 24 h at different concentrations 1 and 10 mg·L^−1^., and ii) to study the accumulation of fluorescent 100 nm PS NPs in the three subpopulations.

The concentrations of PS NPs used in this work represent the highest environmental levels of total MPs floating on the surface of the gyres, however the environmental concentration of NPs is unknown due to analytical limitation. Some studies have demonstrated that the number of plastic particles increase with lower sizes^37^. Therefore, in this study were tested two concentrations of PS NPs, one of them realistic according with MPs concentration and the other one higher environmental concentration due to constant plastic fragmentation.

## Materials and Methods

### Reagents

PS NPs of 50 nm (cat#08691), 100 nm (cat#00876), 100 nm fluoresbrite ex/em: 488/520 nm (cat# 17150-10) and 1 µm (cat#07310) were supplied by Polyscience, Inc.

### Suspensions of NPs

Stock suspensions of PS NPs were prepared in ultrapure water before the experiments. Stocks were sonicated with a tip sonicator (UP 200 S Dr. Hielscher GmbH) for 10 min with pulses each 0.5 seconds and 50% of the maximum amplitude of the tip sonicator.

### Primary and secondary characterization of the PS NPs

Particle size, shape and structure were analysed by Scanning Electron Microscopy (SEM; n:130 images), Transmission Electron Microscopy (TEM; n:130 images) and Fourier-transformed infrared spectroscopy (FTIR). The corroboration of the material is shown in Fig. S2.

The size distribution and zeta potential in the aqueous fluids [ultrapure water (MQ), artificial marine water and serum hemolymph (SH) filtered by 0.2 µm and diluted 1:1 with anti-aggregation solution (171 mM NaCl; 0.2 M Tris; 0.15% v/v HCl 1 N; 24 mM EDTA);^38^] were studied (at 0, 10, 60 and 180 min). These measurements were performed using Dynamic Light Scattering; DLS (Zetasizer Nano ZS90, Malvern Instruments, equipped with software version 7.10) at 1 mg·L^−1^ (Table found in Supplementary Information). Due to the high polydispersity index (PDI) in artificial marine water and serum of hemolymph (PDI < 0.4), the agglomeration stage of the PS NPs was evaluated using Mastersizer 2000, Malvern Instruments at 26 mg·L^−1^ over time 0, 1, 3 and 24 h. Furthermore, to assess structural changes on the surface of the PS NPs, SEM images were performed after 24 h in the different aqueous fluids. Data for ultrapure water and artificial marine water have been published in a previous publication, however these data have been shown to compare with data recorded when PS NPs were suspended in SH^39^.

### Animal, hemolymph collection and in vitro assay hemocyte treatments

*Mytilus galloprovincialis Lam*. specimens 6.5 ± 1 cm of length and 3.2 ± 0.5 cm of width were purchased from an aquaculture farm (Mariscos Antón Fernandez, Spain) and kept for 7 days in an oxygenated and recirculated tank at 16 °C and the mussels were fed daily with *T-ISO* microalgae. The hemolymph was extracted from the posterior adductor muscle of at least 50 organisms using a sterile 1 mL syringe with an 18 G1/2′′ needle.

Once removed from the needle (syringe), the hemolymph was filtered through a sterile filter of 70 µm and pooled at 4 °C with a proportion of 1:1 of anti-aggregation solution.

The three subpopulations of hemocytes were identified by flow cytometry (FSC vs SSC) and cell density was fixed at 2·10^5^ cell·mL^−1^. The samples were diluted with the anti-aggregation solution previously mentioned.

The experiment was carried out with 5 mL of 2·10^5^ cell·mL^−1^ of hemolymph which was incubated at 16 °C in a multiwell plate with 1 and 10 mg·L^−1^ of 50 nm, 100 nm and 1 µm PS NPs. Hemocyte sampling times were after 3 and 24 h of post-exposure to the PS NPs. Short-term toxicity is assessed in the most of *in vitro* assays with primary cell culture due to the viability of the cells. Furthermore, the effects in *in vitro* assays are found more quickly than *in vivo* experiment. Therefore, in this study has been assessed the effects of PS NPs after 3 and 24 h of exposure.

### Flow cytometry analysis

All measurements by flow cytometry were performed with an Accuri C6 flow cytometer with a laser excitation of 488 nm and three channels of emission (band pass (BP) 533/30 nm, band pass (BP) 585/40 nm and long pass (LP) of 670 nm. Furthermore, two more detectors were used, a side scatter detector (SSC) and forward scatter detector (FSC) related to the internal granularity and the volume of cells respectively (Shapiro, 2005). All incubations with fluorophores were performed at 16 °C in darkness.

#### Toxicological responses

##### Non-viable and apoptotic cells

The cell apoptosis assay was performed using the Annexin V-FITC Apoptosis Detection Kit BioVision according to the manufacturer’s instructions. Annexin V-FITC to detect phosphatidylserine in the outside layer of the cytoplasmtic membrane was recorded by an FL1 detector and the Propidium Iodide only able to cross the membrane in dead cells was recorded by an FL2 detector.

##### Mitochondrial membrane potential

Mitochondrial membrane potential was evaluated using the lipophilic cationic probe JC-1 (5,5′,6,6′-tetrachloro-1,1′,3,3′-tetraethyl benzimidazole carbocyanine iodide). Depolarised mitochondrial membrane was recorded using an FL1 detector [band pass (BP) 533/30 nm] (Prado 2012). The samples were incubated with 3 μM of JC-1 for 20 min. To verify the specificity of the JC-1 staining, the cells were treated with carbonyl cyanide m-chlorophenylhydrazone (CCCP) (final concentration of 49 μM during 15 min).

##### Percentage of low DNA content

Syto 13 can cross cytoplasmic membrane and intercalate with nucleic acids stoichiometrically. This fluorophore marks DNA when it is excited with 488 nm. Syto 13 was added at 1 µM and it was incubated for 10 min^40^.

##### Complexity and volume of the cells

Changes in complexity and volume of the different subpopulations of hemocytes were detected through side scatter (SSC) and forward scatter (FSC) detectors of flow cytometry.

#### Immune responses

##### Toxic oxygen radicals

Production of intracellular ROS (reactive oxygen species such as H_2_O_2_, O_2_^.^ and OH^−^) was quantified using the 2′-7′-dichlorofluorescein diacetate (DCFH-DA). A final concentration of DCFH-DA at 80 µM was added to the samples as they incubated for over thirty min. in darkness at room temperature conditions^41^. The ROS present in the cells was measured by an FL1 detector [band pass (BP) 533/30 nm].

H_2_O_2_ was analysed using dihydrorhodamine 1, 2, 3 (DHR 123). Samples were incubated at a final concentration of 28.87 µM and in darkness at room temperature for 60 min. The H_2_O_2_ present in the cells was measured by an FL1 detector [band pass (BP) 533/30 nm].

Hydroethidine (HE) or dihydroethidium has been widely used to detect intracellular O_2_. in cells^42^. Samples were incubated with HE at a final concentration of 15.85 µM and in darkness at room temperature for over 30 min. The O_2_ present in the cells was measured by an FL2 detector [band pass (BP) 585/40 nm]^43^.

The percentage of cells marked with DCFH-DA, DHR 123 and HE was calculated as a proportion of the total cell population.

The positive controls were developed with 0.1 mM of H_2_O_2_ to corroborate the validation methods of the oxidative biomarkers.

##### Toxic nitric radicals

DAF-FM diacetate (D-23842, Molecular probes) is able to quantify low concentrations of intracellular nitric oxide. The fluorescence of DAF-FM is detected using an FL1 detector [band pass (BP) 533/30 nm]. The samples were incubated for 1 h in darkness with a concentration of 10 µM of DAF-FM from a stock prepared in DMSO.

##### Phagocytic capacity

Cells were washed with the anti-aggregation solution, and artificial marine water in a relation 1:1 to remove the PS NPs at 300 g for 10 min. The samples were incubated with 10:1 (100 nm fluoresbrite PS:hemocyte ratio). The incubation time was 2 h. After incubation, uninternalized particles were removed by washing the cells with PBS. Phagocytosis was analysed in R1 by an FL1 detector [band pass (BP) 533/30 nm]. This response was only analysed in R1 due to this subpopulation are professional phagocytes.

##### Tracking of lysosomes

The Lysotracker probe (L7526, Termofisher) is acidotropic and therefore able to keep track of the acid organelles (lysosomes) of living cells. A concentration of 75 nM of lysotracker was added to each sample and incubated in the dark for 2 h, the signal was detected by FL1 detector.

### Statistical analysis

All tests with their controls were performed in triplicate. Data are shown as average±standard deviation (n:3). Statistical analysis was carried out using the IBM SPSS, statistics 23 program. A repeated measures ANOVA analysis was performed to study the differences between times; the times (2 levels) were within-subjects and treatment and concentration were between-subjects. When differences between the times were not observed, the average of the time (3 and 24 h) was calculated and is shown in Figures as a single time. After the study of repeated measures, a one-way ANOVA with a Bonferroni post hoc test was developed for each toxicological and immune response at every sampling time (significant differences at p < 0.05). One-way ANOVA was also performed to study the differences among the hemocyte subpopulations (R1, R2 and R3) according to the variables studied. Normality of data was checked.

In order to provide a description of the structure and distribution of the data without missing any information, a principal component analysis (PCA) was developed to assess the effects of the PS NPs on the toxicological and immune response variables measured in the hemocyte subpopulations.

## Results

### Primary and Secondary characterization of the PS NPs

Nominal size, spherical shape and material composition were in agreement with the suppliers for all the PS NPs tested in this study. When the size distribution of the PS NPs was studied by DLS, the lowest mean values were found for the PS NPs suspended in serum of hemolymph, and the highest mean was found in artificial marine water (Table S1). The zeta potential in all cases was found in an unstable zone between -30-30 mV. It changed from values close to −20 mV in ultrapure water to −5 mV in serum of hemolymph (Table S1).

**Figure 1:**
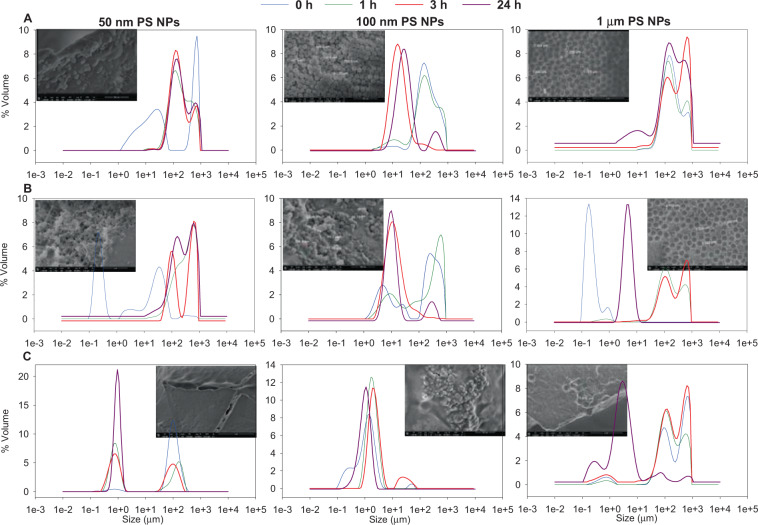
Agglomeration and SEM images of the PS NPs studied. Agglomeration was studied over time (0, 1, 3 and 24 h) in different aqueous suspensions; ultrapure water (panel A), artificial marine water (panel 2) and serum hemolymph, 1:1 serum of hemolymph:anti-aggregation solution (panel C) by Static Light Scattering (SLS), also SEM images of the nanoparticles in the different cultures media were taken after 24 h.

Due to the high PDI in the artificial marine water, serum of hemolymph and 1 µm PS NPs, the agglomeration stage was analysed by SLS (Fig. 1). The three PS NPs used showed a lower size distribution in serum of hemolymph with respect to the other media. The 50 nm and 1 µm PS NPs showed two populations of size distribution from 0 h to 24 h. In addition, all the PS NPs showed a similar behaviour with time, the size distribution of the PS NPs was lower at 24 h with respect to 0 h in artificial marine water and serum of hemoplymh. One µm PS NPs in serum of hemolymph at 24 h was the only PS NPs that maintained the higher percentage in volume of particles according to the nominal size.

The SEM images, after 24 h, in different culture media showed that the PS NPs with greater nominal size conserved the shape and individuality 50 nm <100 nm <1 µm better, while the smaller PS NPs seemed to be aggregates and crimped in a complex matrix (Fig. 1). Furthermore, more complex matrixes such as serum of hemolymph showed changes on the PS NPs surface through SEM images, while in UW the spherical shape was conserved after 24 h for all the PS NPs (Fig. 1).

### Toxicological responses

**Figure 2:**
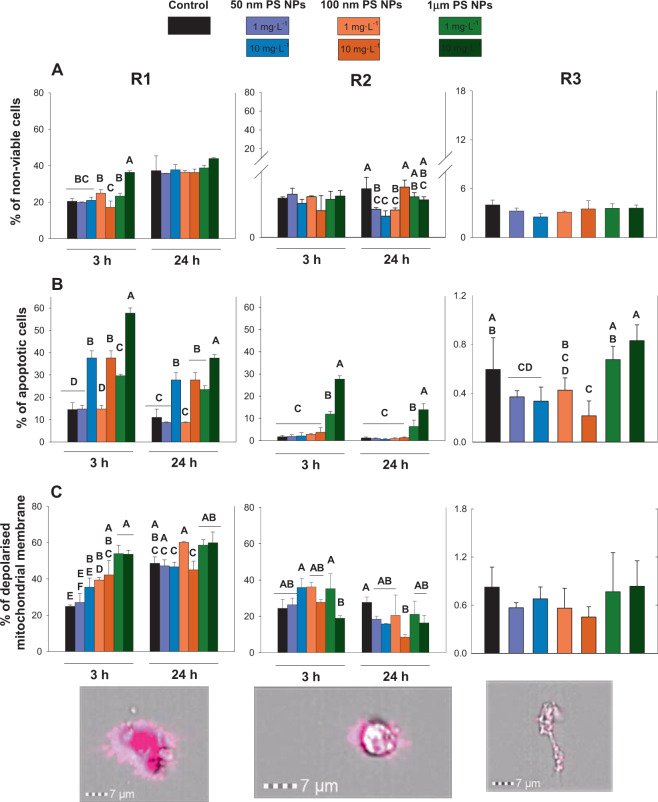
Toxicological responses (non-viable cells, apoptotic cells and depolarised mitochondrial membrane; panels A, B and C respectively) measured at 3 and 24 h when R1, R2 and R3 were exposed to 1 and 10 mg·L^−1^ of 50 nm, 100 nm and 1 µm PS NPs. Different uppercases represent significant differences (p < 0.05; Bomberroni’s post hoc test; n:3) among the treatments and concentrations tested. No significant differences between times by Repeated Measured GLM analysis are shown as a single graph (mean ± SD between both times). Amnis imaging flow cytometers are shown.

With regard to cell viability, R1 was the most affected hemocyte subpopulation reaching up to values higher than 20% of non-viable cells; on the other hand, R2 and R3 did not reach this percentage. Therefore, significant differences between R1 and R2/R3 for this response were observed (p < 0.05; Fig. 2A; Table S2). Focusing on R1, the differences in the non-viable cells between the controls and treatments were only found after 3 h of exposure for 1 µm at 10 mg·L^−1^ with values of 36.3 ± 0.7% of non-viable cells (p < 0.05; Fig. 2A).

Concerning the apoptotic cells, the R1 subpopulation showed significant differences with respect to the other regions R2/R3 (p < 0.05; Fig. 2B), reaching values higher than 57.7 ± 2.3% of apoptotic cells. The subpopulations R1 and R2 were significantly affected when the hemocytes were exposed to both concentrations of 1 µm PS NPs, but additionally R1 was also affected by 100 nm PS NPs at 10 mg·L^−1^ (p < 0.05; Fig. 2B).

In line with the previous responses, the depolarised mitochondrial membrane in R1 showed the same pattern when it was compared to the other subpopulations R2/R3 (p < 0.05; Fig. 2C), although R2 and R3 likewise showed differences between them. R1 exposed to 100 nm and 1 µm PS NPs increased significantly with respect to the controls at 3 h (p < 0.05). Drawing a comparison between the controls and treatments for this response, the highest concentration of 100 nm PS NPs and both concentrations of 1 µm PS NPs showed significant differences when R1 was exposed to the previously mentioned treatments (p < 0.05; Fig. 2C). The R2 population behaved differently (Fig. 2C). The R2 population exposed to PS NPs showed a decreased trend in depolarised mitochondrial membrane after 24 h; however, significant effects were only shown under 10 mg·L^−1^ of 100 nm PS NPs (p < 0.05; Fig. 2C).

After 3 h of PS NPs exposure, the effects in percentage of apoptotic cells and depolarized mitochondrial membrane were higher than the effects after 24 h being the time statistically significant (p < 0.05)for R1 and R2 hemocytes population. On the other hand R3 did not show differences between times for these responses (Fig. 2).

**Figure 3:**
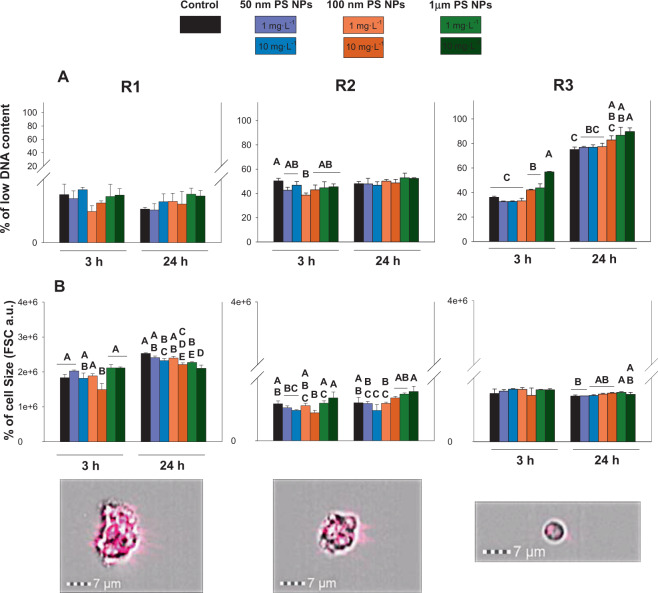
Toxicological responses (low DNA content and cell size; panels A and B respectively) measured at 3 and 24 h when R1, R2 and R3 were exposed to 1 and 10 mg·L^−1^ of 50 nm, 100 nm and 1 µm PS NPs. Different uppercases represent significant differences (p < 0.05; Bomberroni’s post hoc test; n:3) among the treatments and concentrations tested. Amnis imaging flow cytometers are shown.

Contrary to the previous responses studied, R3 was the subpopulation that showed heterogeneity in the DNA content. Two populations were found in R3, one of them with a high DNA content and another with a low DNA content. R1/R2 showed a more homogeneous DNA content without a significant increase in the low DNA content region. R3 displayed significant differences in regard to R1/R2 (p < 0.05; Fig. 3A). Focusing on R3, significant differences between the controls and both concentrations of 1 µm PS NPs were detected at 3 and 24 h (p < 0.05). Furthermore, the higher differences between times (3 and 24 h) were found for the R3 hemocytes population.

In relation to inherent cell properties such as cell size, R1, once again, was the most affected subpopulation (Fig. 3B,C). With respect to cell size, R1 showed lower values in this response when cells were exposed to both concentrations of 100 nm and 1 µm PS NPs in relation to the controls at 24 h (p < 0.05; Fig. 3B). Cell size showed significant differences between times (3 and 24 h) for each hemocytes population, however, R1 was the population with higher changes between controls and PS NPs treatments. No relevant results were found when cell complexity was studied (Fig. S3).

### Immune responses

#### Toxic oxygen radicals

**Figure 4:**
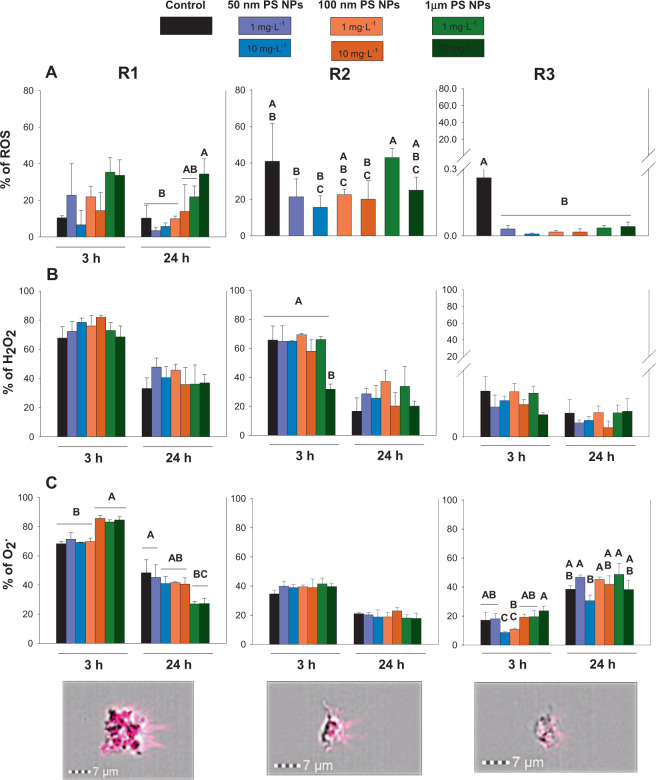
Oxygen toxic radicals (ROS, H_2_O_2_ and O_2_^.^; panel A, B and C respectively) measured at 3 and 24 h when R1, R2 and R3 were exposed to 1 and 10 mg·L^−1^ of 50 nm, 100 nm and 1 µm PS NPs. Different uppercases represent significant differences (p < 0.05; Bomberroni’s post hoc test; n:3) among the treatments and concentrations tested. No significant differences between times by Repeated Measured GLM analysis are shown as a single graph (mean ± SD between both times). Amnis imaging flow cytometers are shown.

According to the data regarding ROS, the R1 and R2 subpopulations showed higher values in % of ROS than R3, therefore significant differences between them were observed (p < 0.05; Fig. 4A; Table S2). However, R1 and R3 were the regions which showed significant differences between the controls and treatments, although with a different pattern. The trend of R1 was to increase the level of ROS under 10 mg·L^−1^ of 1 µm PS NPs, nevertheless, the level of ROS in all treatments in R3 decreased significantly with respect to the controls.

In relation to the percentage of O_2_., R1 was different from R2/R3 (p < 0.05, Fig. 4B). R1 was the only subpopulation affected by the PS NPs when the cells were exposed to both concentrations of 1 µm PS NPs at 3 and 24 h (p < 0.05).

R1 and R2 followed the same trend; a higher level of O_2_. and H_2_O_2_ at 3 h with respect to the levels found at 24 h. The contrary was found for R3 in the case of O_2_., where the levels of O_2_. were higher at 24 h than the levels at 3 h.

Concerning the data related to H_2_O_2_ levels, the three subpopulations showed differences (p < 0.05). Significant differences between the controls and treatments were only detected in R2 at 3 h in the case of 1 µm PS NPs at 10 mg·L^−1^ (p < 0.05, Fig. 4C).

Percentage of H_2_O_2_ and O_2_. showed differences between times (3 and 24 h) for the three population studied. R1 and R2 showed higher values of H2O2 and O_2_. after 3 h than 24 h of PS NPs exposure.

#### Toxic nitric radicals

**Figure 5:**
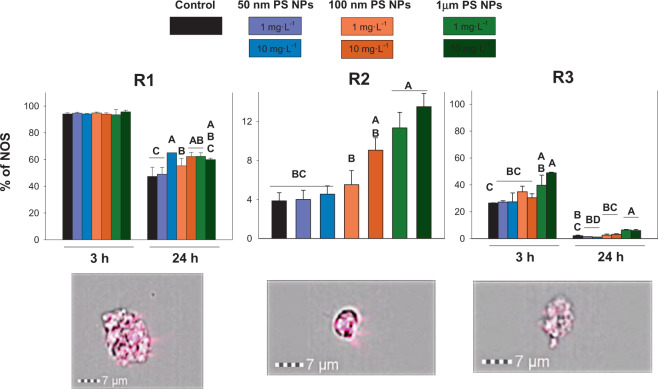
Nitric toxic radicals measured at 3 and 24 h when R1, R2 and R3 were exposed to 1 and 10 mg·L^−1^ of 50 nm, 100 nm and 1 µm of PS NPs. Different uppercases represent significant differences (p < 0.05; Bomberroni’s post hoc test; n:3) among the treatments and concentrations tested. No significant differences between times by Repeated Measured GLM analysis are shown as a single graph (mean ± SD between both times). Amnis imaging flow cytometers are shown.

According to the changes in the percentage of NOS, R1, R2 and R3 showed significant changes in their levels of NOS with respect to the controls. Each population was different concerning the levels of NOS (p < 0.05; Fig. 5; Table S2). R2 and R3 showed a significant increase in % of NOS for all sampling times studied when comparing the controls and both concentrations of 1 µm PS NPs. However, R1 showed significant differences between the controls and both concentrations of 50 and 100 nm PS NPs (p < 0.05; Fig. 5). Population R1 and R3 showed differences between times (3 and 24 h). After 24 h of exposure were found lower values in percentage of NPS than values found after 3 h of exposure. Although this values of NOS were lower after 24 h, in this time were found the higher differences between controls and PS NPs treatments.

#### Phagocytic capacity

**Figure 6:**
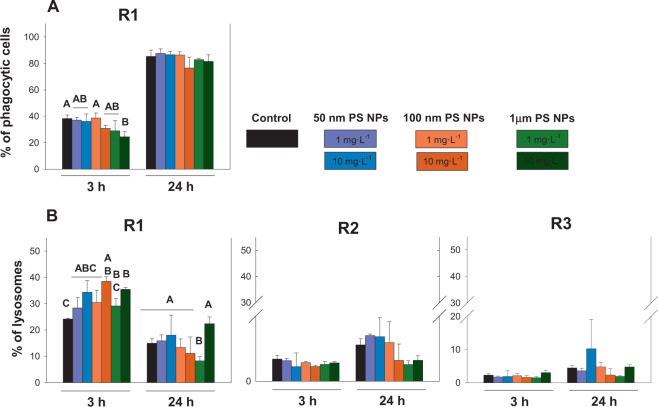
Phagocytic capacity and percentage of Lysosomes (panel A and B respectively) measured at 3 and 24 h when R1, R2 and R3 were exposed to 1 and 10 mg·L^−1^ of 50 nm, 100 nm and 1 µm PS NPs. Different uppercases represent significant differences (p < 0.05; Bomberroni’s post hoc test; n:3) among the treatments and concentrations tested.

Phagocytic capacity was studied for R1 in this work to be the main cell subpopulations specialized in this function. Differences between times were found for phagocytic capacity, showing lower values after 3 h of exposure than after 24 h. However, a significant decrease in phagocytic capacity was found between the controls and 10 mg·L^−1^ of 1 µm PS NPs at the first sampling time (3 h); (p < 0.05; Fig. 6A).

#### Tracking of lysosomes

Significant differences between R1 and R2/R3 were observed (p < 0.005, Table S2) when tracking the lysosomes. Differences between 3 and 24 h were found in R1, R2 and R3. R1 showed lower lysosomes marked at 24 h while R2 showed lower values in lysosomes marked after 3 h (Fig. 6B). R1 was the only subpopulation that showed differences in the number of lysosomes between the controls and treatments (Fig. 6B). At 3 h an increase in the number of lysosomes were found between the controls and 10 mg·L^−1^ of 100 nm and 1 µm of PS NPs (p < 0.05). However, at 24 h a significant decrease in the number of lysosomes was found at 1 mg·L^−1^ of 1 µm PS NPs with respect to the controls (p < 0.05).

## Discussion

### The importance of developing ecotoxicological tests in biological fluids

The approach that includes developing a toxicological test in biological fluids could provide relevant insights about the behaviour, bioavailability and toxicity of NPs and MPs. All the PS NPs, independently of their nominal size, showed two populations of PS NPs size after 3 h in hemolymph serum using the SLS technique. Furthermore, the size distribution of all the PS NPs in the hemolymph serum showed the smallest size compared to the PS NPs suspended in ultrapure water and artificial marine water. Some works have identified protein-corona in NPs when they were suspended in biological fluids^44^. The acquired secondary properties of NPs define a new biological identity of the NPs and therefore the interaction with their surrounding environments^45^. Hemolymph serum of *Mytilus galloprovincialis* is characterized by abundant proteins such as extrapallial protein (EP) precursor, astacin and Cu-Zn superoxide dismutase^46^. The protein-corona is important in defining the surface properties, charges, resistance to aggregation and hydrodynamic size of nanoparticles^30^. In the work of Canesi *et al*. (2017), negative zeta potential was key in the formation of the protein-corona in CeO_2_ NPs when they were suspended in hemolymph serum^44^. In our study, the charge of the PS NPs descended drastically in the hemolymph serum for the PS NPs tested with respect to the PS NPs suspended in ultrapure water. This same pattern was found in the literature for CeO_2_ and TiO_2_ PS NPs where these NPs also formed protein-corona, although the protein-corona for metallic nanoparticles and NH_2_-PS NPs were different. The protein-corona of metallic nanoparticles was Cu, Zn SOD and the protein-corona of NH_2_-PS NPs was a Putative C1q domain containing protein (MgC1q6)^44,47,48^. Recently, in the work of Détrée and Gallardo-Escárate (2018) the Putative C1q gene family was upregulated in the single and repetitive microplastic exposure^48^.

The new PS NP properties, such as protein-corona, possess long-term stability in biological environments, and thus reduce the free energy of the NPs but, on the other hand, they may interact with membrane receptors, particle wrapping, trafficking, cellular uptake, pattern of biodistribution and immune responses^33,49^. The original shape of all the particles was spherical; however, the spherical shape was unclear when the aqueous matrix was more complex. For instance, in the hemolymph serum, the surface particles showed surface irregularities, this was more discernible for the particle surface of 1 µm PS NPs with respect to the particles suspended in ultrapure and artificial seawater.

All the PS NPs with a different nominal size after 24 h showed the same size distribution with a size around 1 µm in the hemolymph serum, therefore the aggregation/agglomeration for the different PS NPs were different. Currently, when differences between aggregates and agglomerates are not possible to determine, the term of “cluster” is the most common term employed^53^. The PS NPs of 50 and 100 nm showed greater clusters than the 1 µm PS NPs. Clusters of NPs are governed by the primary characteristics of the NPs “per se” and the secondary characteristics of media such as pH, ionic strength and organic matter^54^. Although the ionic strength of biological fluids in marine organisms is similar to seawater, the behaviour of the PS NPs in this study was different. Therefore, the authors suggest developing *in vitro* assays in the biological fluids themselves or with minimum modifications (eg. An antiaggregation solution).

SEM images of the PS NPs in different culture media showed that the 1 µm PS NPs were able to conserve their spherical form, individuality and original features with respect to the 50 and 100 nm PS NPs after 24 h. The differences in nominal size among the PS NPs used in this work could determine the colloidal forces among the particles. Different colloidal forces join at the interface NPs-biological fluids according to the NPs size and media characteristics^50^. The forces which govern the interactions of small particles (<100 nm) with a greater surface area and reactive energy are polymer bridging, steric, solvent, electrostatic and electrodynamic interactions, whereas the governing forces in particles bigger than 100 nm are hydrodynamic interactions^30^. Therefore, due to the SEM images and size distribution, the authors suggest that the 1 µm PS NPs were governed by different colloidal forces than the 50 and 100 nm PS NPs, making the 1 µm PS NPs more stable and bioavailable to the hemocytes. Our results concerning the toxicological results and effects on the immune responses support this hypothesis. The toxicological responses such as: non-viable cells, apoptotic cells, depolarised mitochondrial membrane, DNA damage and cell size in mussel hemocytes proved to be more sensitive when they were exposed to the 1 µm PS NPs than the 50 and 100 nm PS NPs. Furthermore, immune responses such as toxic oxygen radicals, toxic nitrogen radicals, phagocytic capacity and tracking of lysosomes showed the same pattern when hemocytes were exposed to 1 µm PS NPs.

### Sensitivity of hemocyte subpopulations to PS NPs exposure

**Figure 7:**
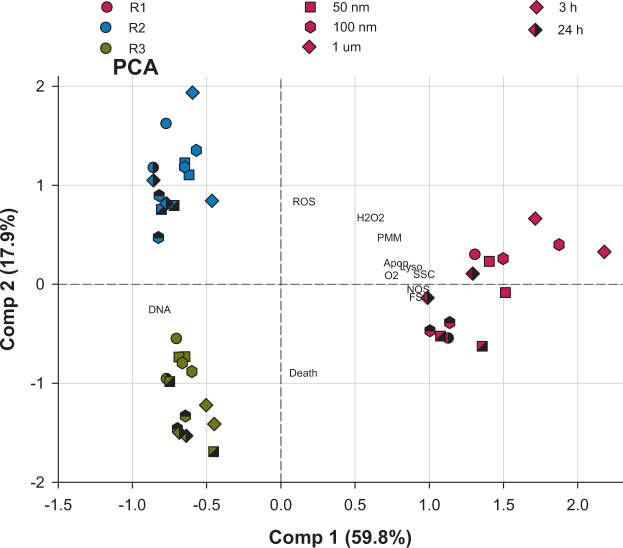
Principal components (1 and 2) of the toxicological and immune responses measured after 3 and 24 h in R1, R2 and R3 when the hemocytes were exposed to 1 and 10 mg·L^−1^ of 50 nm, 100 nm and 1 µm PS NPs.

Hemocytes are cells that mediate immunity, these phagocytic cells are specialized in several immunomodulation strategies to fight against foreign bodies and they are involved in digestion, shell repair, respiration, osmoregulation, transport and excretion. Hence, in literature hemocytes are designated as primary target of MPs and NPs in freshwater and marine organisms^51^. Therefore, currently, immunological responses have been used to assess the health status of bivalves exposed to metallic nanoparticles^58–64^. In most of these studies, hemocytes are considered a set of cells; however, it is known that different cell subpopulations are found. The hemocyte subpopulations are specialist and perform specific skills and tasks in host defence^36^. Flow cytometry is a reliable tool to determine hemocyte subpopulations through size and cell complexity. Different ecotoxicological and immunological responses were studied in three subpopulations defined according to García-García *et al*. (2008) and Le Foll *et al*. (2010) in this study. These were a first population of large-granular cells (R1), a second population of small-semigranular cells (R2) and a third population of hyaline cells (R3). R1 showed significant changes in the immune responses measured in this study with respect to a lower immune modulation of R2 and R3. Distribution of the data responses including the three hemocyte subpopulations are shown through a PCA analysis in Fig. 7. The first component describes the highest percentage of the total variance (59.8%), while the second component explains 17.9% of the total variance. In the PCA the first component is explained by: O_2_·, NOS, tracking lysosomes, apoptosis, depolarised mitochondrial membrane, DNA damage, cell size and cell complexity, while the second component is explained by non-viable cells, ROS and H_2_O_2_ (Fig. 7). The clustering of R1 was driven by O_2_·, NOS, tracking lysosomes, apoptosis, depolarised mitochondrial membrane, ROS, H_2_O_2_, cell size and cell complexity, while the clustering of R3 was driven by DNA damage.

It is obvious that *M. galloprovincialis* hemocytes are cells for special purposes. R1 granulocytes are the phagocytic expert defined in literature, and R1 was the subpopulation more involved in the immunomodulation against PS NP exposure while R2 and R3 appear to play a less important role in the immune responses measured. Some authors suggest that hyaline cells may join in other immune functions such as coagulation, encapsulation, or even defence against viruses^36,52^.

Phagocytosis is the primary and most efficient mechanism for the killing of bacteria, elimination of foreign particles in these organisms and maintaining homeostasis^53^. As phagocytosis is the main defence mechanism for bivalves, a decrease in phagocytic activity may have a detrimental effect, producing an alteration in food ingestion, energy assimilation processes, ability to remove pathogens, and the overall viability of cells^54^. 1 µm PS NPs at 10 mg·L^−1^ were the only particles which provoked a significant decrease in phagocytic activity. This is not surprising since 1 µm PS NPs were the particles which triggered higher responses in the immunological variables measured in this work. Several studies have demonstrated that some metallic nanoparticles can inhibit the phagocytosis in *M. galloprovincialis* in “*in vitro”* studies^55–57^. *In vitro* assays developed with nanoplastics and *M. galloprovincialis* hemocytes have also corroborated a decrease in phagocytic activity when hemocytes were exposed to PS-NH_2_ NPs from 1 mg·L^−1^ ^32,33^.

The uptake of 100 nm PS NPs was evidenced by flow cytometry and confocal images. Although 100% of the cells in each population showed fluorescence due to 100 nm fluoresbrite PS NPs, this particle did not show the highest response with respect to the 50 nm and 1 µm PS NPs. Phagocytosis is frequently assumed to be an uptake of particles bigger than 0.05 µm; however, there are other mechanisms of uptake into cells for particles smaller than 0.05 µm that include: passive diffusion, transport through ion channels, carrier-mediated transport, phagocytosis, micropinocytosis, macropinocytosis and caveolar/endocytic routes^58^. Endocytic pathways lead to NPs though intracellular vesicles including early and late endosomes and lysosomes, or phagosome endosomal and lysosomal compartments^59^. Lysosomes are the main and sensitive compartments for intracellular sequestration of NPs and detoxification^20,59–61^. In the present study, an increase in lysosomes and/or autophagolysosome accumulation was found. In agreement with our results, an increase in the fluorescence of lysotracker was found when LLC-PK1 cells were exposed to fullenerol NPs as in the study of Johnson-Lyles *et al*.^62^. The lysosomal compartment is necessary for cellular homeostasis and it plays a main role in the cell clearance, energy metabolism and cell signalling involved in functions such as innate immunity, calcium signalling, or apoptosis^63^. Related to apoptosis, the R1 and R2 hemocyte subpopulations showed signs of apoptosis in the cytoplasmic membrane; however, R1 was the population with a depolarization of the mitochondrial membrane while in R2 this depolarization was not observed. The authors of the present study suggest different pathways in the apoptosis process for R1 and R2, an intrinsic route for R1 in contrast with an extrinsic route for the R2 population. In the intrinsic route of apoptosis, ROS are involved in the oxidation of mitochondrial pores for the release of cyto-c^64^. Therefore, the increase in the intracellular ROS level reported in this work could also have been generated by an intrinsic route for the apoptotic process. On the other hand, the 100 nm PS NPs provoked a significant decrease in mitochondrial membrane potential in R2 as well as in the study by Canesi *et al*. (2015), when *M. galloprovincialis* hemocytes were exposed to 100 mg·L^−1^ of PS-NH_2_^36^. The MPs, in an *in vivo* study performed with *M. galloprovincialis* exposed to 1.5 g·L^−1^ PS and PE in a scale range of 1000–100 µm demonstrated significant accumulation and changes in hemocytes such as a decrease in phagocytic activity, stability in lysosome membrane and granulocyte/hyalinocyte ratio^65^.

### Importance of PS NPs size and time in immune responses

Despite the size of the PS NPs tested being in the nanoplastic size range (<1 µm), the behaviour (agglomeration stage and conservation of origin spherical shape) in the same biological fluid (hemolymph serum) was different, as explained in section 4.1. These differences were also found in the immunological responses measured in the hemocytes exposed to different sizes of PS NPs. The immunoregulation responses were related to the PS NPs size, with the larger causing greater effects. The relation between the size of the PS NPs and their effects on the hemocytes is a fact; however, the reason why bigger PS NPs triggered greater effects than the smaller PS NPs is currently unknown. Furthermore, time showed to play a key role in the immune responses measured, however any trend was found at 3 and 24 h and it was dependent of each endpoints. On the other hand, differences between times (3 and 24 h) were found some responses such as percentage in H_2_O_2_, O_2_^.^, NOS, phagocytic capacity and apoptotic cells. The mentioned responses found higher levels after 3 h than at 24 h. This decreasement could be due to high efficiency of hemocytes in cleaning its surrounding environment. Therefore, viable hemocytes could be phagocytizing apoptotic, necrotic and debris of cells. In the Fig. S4 is showed the increase of the percentage of R1 cell population after 24 h respect to 3 h of experiment, therefore the hypothesis about phagocytic capacity of this population to clean damage cells of the culture media is supported.

## Conclusions

Although many studies have evidenced the presence of plastic litter in the ocean and its degradation into smaller fragments (MPs and NPs), analyses concerning the detection of and levels of concentration for NPs are not yet available. However, the presence of MPs and their degradation is a fact. Knowing the effects that NPs have on susceptible or target marine animals is essential, both now and for the future. The innate immune system of bivalves is considered to be a sentinel concerning knowledge related to the effects of nanomaterials. The intrinsic characteristic of nanoplastics and the surrounding environment in which they are found (such as hemolymph serum) can determine secondary identity according to agglomeration, the new shape acquired and protein corona formation. It was found that 1 µm PS NPs, due to their size, were the most stable PS NPs in hemolymph serum in terms of agglomerate size and shape compared to the 50 and 100 nm PS NPs in this study. These nanoplastics (1 µm PS NPs) provoked the greatest effects on the immune system of *M. galloprovincialis*, possibly due to the behaviour of these particles in hemolymph. The R1 subpopulation displayed the clearest immunomodulation processes when the immune system was exposed to PS NPs, showing greater changes in the immune responses measured compared to the R2 and R3 hemocyte populations. In addition, the internalization of 100 nm fluoresbrite PS NPs was confirmed by flow cytometry and confocal images.

## Supplementary information

Supplementary information.
