# Supplementary material for: Immunotoxicity of polystyrene nanoplastics in different hemocyte subpopulations of Mytilus galloprovincialis
Source: Sci Rep. 2020 May 25;10:8637. doi: 10.1038/s41598-020-65596-8 (PMC7248110; doi:10.1038/s41598-020-65596-8)
Supplement: Supplementary file 1 — Supplementary information. [file 41598_2020_65596_MOESM1_ESM.docx]

**Immunotoxicity of polystyrene nanoplastics in different hemocyte subpopulations of *Mytilus galloprovincialis*.**

**Sendra, Marta^1,2^; Carrasco-Braganza, María Isabel ^2^; Yeste, Pilar María^3^; Vila, Marta^4^ & Blasco, Julián^2^.**

^1^ CSIC, Spanish National Reference Laboratory for Mollusc Diseases, Instituto de Investigaciones Marinas, 36208 Vigo, Spain

^2^ Department of Ecology and Coastal Management, Institute of Marine Sciences of Andalusia (CSIC), Campus Río San Pedro, 11510 Puerto Real, Cádiz, Spain

^3^ Department of Material Science, Metallurgical Engineering and Inorganic Chemistry, University of Cádiz, Spain.

^4^ Laboratory of Biochemistry and Molecular Biology. University of Huelva, Spain.

**Author for correspondence:** [**marta.sendra@icman.csic.es**](mailto:marta.sendra@icman.csic.es)

**Figure S1**. Flow cytometry dot plot of the different hemocyte populations. Axes X and Y show cell size (FSC) and cell complexity (SSC) respectively. R1 shows large granular cells, R2 small semigranular cells and R3 small agranular or hyaline cells. These regions and categories were determined following the literature and confirmed by Amnis® imaging flow cytometers (these pictures were added at the end of the Figures).


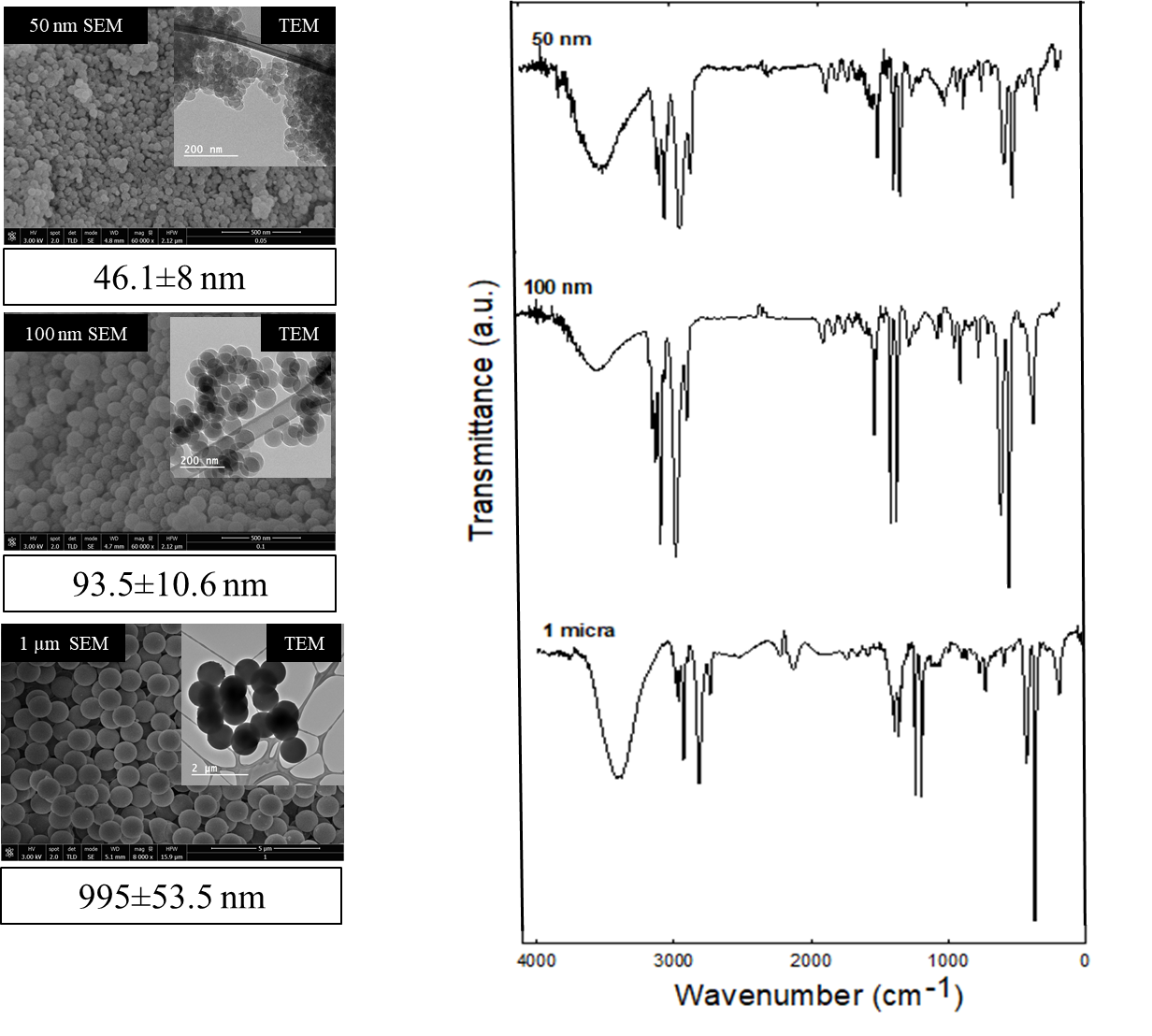


**Figure S2.** Primary characterization of the PS NPs. TEM and SEM of PS NPs (130 particles were analysed to determine average size and standard deviation). FTIR analysis. The infrared spectrum bands correspond to the functional groups of the polystyrene molecule 3081.2 cm^-1^ , -3001.11 cm^-1^ C-H aromatic tension, 2923.91 cm^-1^ and 2850.40 cm^-1^ CH_2_ asymmetric and symmetric tension; 1943.19 –1728.23 cm^-1^ aromatic ring monosubstitution 1452.28 cm^-1^ deformation CH_2_ + C=C of the aromatic ring 1069.65  cm^-1^ ﬂexion C-H in the plane.

**Figure S3.** Cell complexity measured at 3 and 24 h when R1, R2 and R3 were exposed to 1 and 10 mg·L^-1^ of 50 nm, 100 nm and 1 µm of PS NPs (mean±SD between both times). Different uppercases represent significant differences (p<0.05; Bonferroni’s post hoc test; n:3) among the treatments and concentrations tested.

**Figure S4.** Percentage of R1 cells respect to the total cell population. Capital letters show the significant differences between treatments and asterisks show the differences between 3 and 24 h in the same treatment (p<0.05).

**Table S1.** Secondary characterization (mean and peak size of agglomerates, polydispersity index (PDI) and zeta potential (**ζ**) by DLS in ultrapure, artificial marine water and serum of hemolymph (1:1, serum of hemolymph:anti-aggregation solution). Data for ultrapure water and artificial marine water from Sendra et al., 2019.

| **PS NPs** | **DLS (nm)** | | | | | | | | | | | |
| --- | --- | --- | --- | --- | --- | --- | --- | --- | --- | --- | --- | --- |
|  | **Ultrapure water** | | | | **Artificial marine water** | | | | **Hemolymph serum** | | | |
|  | **Mean (nm)** | **PDI** | **Peak**  **(nm)** | **ζ (mV)** | **Mean (nm)** | **PDI** | **Peak**  **(nm)** | **ζ (mV)** | **Mean (nm)** | **PDI** | **Peak**  **(nm)** | **ζ (mV)** |
| **50 nm** | 151.7 | 0.3 | 113.1±38.3 | -23.9±0.9 | 1426 | 1 | 431.2±31.1 | -5.2±0.04 | 83.3 | 0.4 | 72.4±34.0 | -5.1±0.02 |
| **100 nm** | 121.0 | 0.2 | 106.4±18.1 | -18.8±0.8 | 914.5 | 0.8 | 406.1±41.8 | -12.3±0.3 | 90.8 | 0.4 | 86.2±31.7 | -5.9±0.7 |
| **1 µm** | 1601 | 1 | 199.7 | -22.1±0.3 | 1572 | 1 | 401.9 | -14.6±0.4 | 123.8 | 0.36 | 188.2±60.0 | -5.3±0.28 |

Peak values higher than 30% of the population are shown in this table.

**Table S2.** Differences among hemocyte subpopulations according to the responses measured. ANOVA analysis with a Bonferroni post hoc was performed for both sampling times (3 and 24 h). Significant differences are shown as asterisks (p<0.05).

|  |  | **ROS** | | | **O_2_** | | | **H_2_O_2_** | | | **NOS** | | | **DEATHS** | | | **APOP** | | | **PMM** | | |
| --- | --- | --- | --- | --- | --- | --- | --- | --- | --- | --- | --- | --- | --- | --- | --- | --- | --- | --- | --- | --- | --- | --- |
|  |  | **R1** | **R2** | **R3** | **R1** | **R2** | **R3** | **R1** | **R2** | **R3** | **R1** | **R2** | **R3** | **R1** | **R2** | **R3** | **R1** | **R2** | **R3** | **R1** | **R2** | **R3** |
| **3 h** | **R1** |  | - | * |  | * | * |  | * | * |  | * | * |  | * | * |  | * | * |  | * | * |
|  | **R2** | - |  | * | * |  | * | * |  | * | * |  | * | * |  | * | * |  | - | * |  | * |
|  | **R3** | * | * |  | * | * |  | * | * |  | * | * |  | * | * |  | * | - |  | * | * |  |
|  |  |  |  |  |  |  |  |  |  |  |  |  |  |  |  |  |  |  |  |  |  |  |
| **24 h** | **R1** |  | * | * |  | * | - |  | * | * |  | * | * |  | * | * |  | * | * |  | * | * |
|  | **R2** | * |  | * | * |  | * | * |  | * | * |  | - | * |  | * | * |  | - | * |  | * |
|  | **R3** | * | * |  | - | * |  | * | * |  | * | - |  | * | * |  | * | - |  | * | * |  |

|  |  | **DNA** | | | **LYSO** | | | **SSC** | | | **FSC** | | | **PHAGO** | | | **BIOAC** | | |
| --- | --- | --- | --- | --- | --- | --- | --- | --- | --- | --- | --- | --- | --- | --- | --- | --- | --- | --- | --- |
|  |  | **R1** | **R2** | **R3** | **R1** | **R2** | **R3** | **R1** | **R2** | **R3** | **R1** | **R2** | **R3** | **R1** | **R2** | **R3** | **R1** | **R2** | **R3** |
| **3 h** | **R1** |  | * | * |  | * | * |  | * | * |  | * | * | * |  |  |  | - | - |
|  | **R2** | * |  | * | * |  | - | * |  | * | * |  | * |  |  |  | - |  | - |
|  | **R3** | * | * |  | * | - |  | * | * |  | * | * |  |  |  |  | - | - |  |
|  |  |  |  |  |  |  |  |  |  |  |  |  |  |  |  |  |  |  |  |
| **24 h** | **R1** |  | * | * |  | * | * |  | * | * |  | * | * | * |  |  |  | - | - |
|  | **R2** | * |  | * | * |  | - | * |  | * | * |  | * |  |  |  | - |  | - |
|  | **R3** | * | * |  | * | - |  | * | * |  | * | * |  |  |  |  | - | - |  |

**Table S3.** Contribution of the responses to the dimensions 1 and 2 of the Principal Components Analysis (PCA).

| **Responses** | **Components** | |
| --- | --- | --- |
|  | **Comp. 1** | **Comp. 2** |
| **Cell complexity** | 0.964 | 0.099 |
| **Cell size** | 0.934 | -0.130 |
| **NOS** | 0.925 | -0.053 |
| **Lysosomes tracking** | 0.877 | 0.168 |
| **Low DNA content** | -0.817 | -0.256 |
| **Apoptotic cells** | 0.784 | 0.215 |
| **O_2_^.^** | 0.744 | 0.088 |
| **Mitochondrial membrane potential** | 0.731 | 0.470 |
| **Non-viable cells** | 0.151 | -0.896 |
| **ROS** | 0.156 | 0.832 |
| **H_2_O_2_** | 0.604 | 0.671 |
